# Cup-Shaped Superparamagnetic Hemispheres for Size-Selective Cell Filtration

**DOI:** 10.1038/srep06362

**Published:** 2014-09-15

**Authors:** Hyonchol Kim, Hideyuki Terazono, Hiroyuki Takei, Kenji Yasuda

**Affiliations:** 1 Kanagawa Academy of Science and Technology, KSP East 310, 3-2-1 Sakado, Takatsu-ku, Kawasaki, Kanagawa 213-0012, Japan; 2 Department of Biomedical Information, Division of Biosystems, Institute of Biomaterials and Bioengineering, Tokyo Medical and Dental University, 2-3-10 Kanda-Surugadai, Chiyoda-ku, Tokyo 101-0062, Japan; 3 Department of Life Sciences, Faculty of Life Sciences, Toyo University, 1-1-1 Izumino, Itakura-machi, Oura-gun, Gunma 374-0193, Japan

## Abstract

We propose a new method of size separation of cells exploiting precisely size-controlled hemispherical superparamagnetic microparticles. A three-layered structure of a 2-nm nickel layer inserted between 15-nm silicon dioxide layers was formed on polystyrene cast spheres by vapor deposition. The polystyrene was then removed by burning and the hemispherical superparamagnetic microparticles, “magcups”, were obtained. The standard target cells (CCRF-CEM, 12 ± 2 μm) were mixed with a set of different sizes of the fabricated magcups, and we confirmed that the cells were captured in the magcups having cavities larger than 15 μm in diameter, and then gathered by magnetic force. The collected cells were grown in a culture medium without any damage. The results suggest that this method is quick, simple and non-invasive size separation of target cells.

Purification depending on size is one of the fundamental processes of cell purification especially when the specific molecular biomarker is not identified for the candidate cells. For example, differences in the size of cancer cells from that of normal blood cells were identified, and technologies for isolating such cancer cells from blood depending on their size were developed^1^,^2^,^3^,^4^,^5^,^6^. Membrane filtration is most widely used for such separation because of its preciseness and simple preparation, called ISET (isolation by size of epithelial tumor cells)^1^,^2^. However, it exerts strong shear stress on cells, producing damage, and clogging of membrane pores also reduces the efficiency of filtration. Another popular approach for target cell purification is the use of magnetic particles, called MACS (magnetic cell separation)^7^,^8^,^9^,^10^,^11^,^12^. In this method, target cells are recognized by their specific antibodies immobilized on the magnetic particle surfaces, and collected by magnetic field application. The magnetic particles are dispersed in solvent and attached to target cells; therefore, this method can avoid the shear stress damage to cells upon use of the filtration method; however, target cells should be recognized with probe molecules such as antibodies, and size-dependent cell separation has been difficult using the conventional MACS method.

Here, we propose a new method of size-selective target cell separation that avoids shear stress by using precisely size-controlled hemispherical superparamagnetic microparticles, “magcups”.

## Results

**Figure 1:**
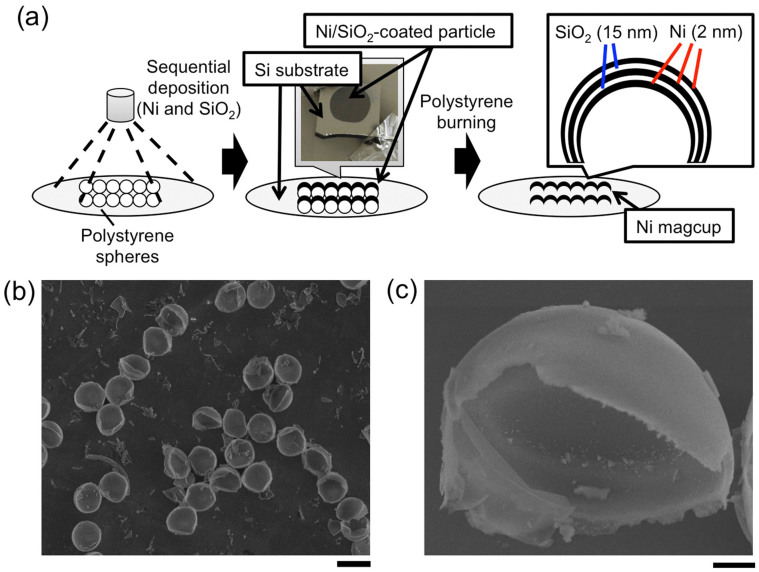
Fabrication of the magcup. (a) Schematic images of the fabrication. First, Ni and SiO_2_ were sequentially deposited on the polystyrene beads at thicknesses of 2 and 15 nm 3 and 2 times, respectively. Next, the deposited polystyrene beads were burned in an electric furnace to remove polystyrene casts from the particles. Then, the magcups composed of Ni and SiO_2_ layers were fabricated. (b, c) FE-SEM images of fabricated magcups at low (b) and high (c) magnifications. Cup size, 10 μm. Bars are 10 μm (b) and 1 μm (c).

We have fabricated precisely size-controlled hemispherical superparamagnetic microparticles (“magcups”) having three layers, namely, a 2-nm nickel layer inserted between 15-nm silicon dioxide layers by vapor deposition. Figure 1 (a) shows an overview of the fabrication of a magcup. The fabrication procedure is fundamentally based on a method described in previous reports^13^,^14^,^15^,^16^,^17^,^18^; specifically, in this study, we precisely controlled the thickness of nickel (Ni) up to 2 nm and the spacing silicon dioxide (SiO_2_) thickness larger than 15 nm to maintain the superparamagnetic conditions. For the magnetic layers, other elements such as iron or cobalt can be also used as suitably, and in this study, Ni was chosen because of its stability in solvents containing high concentration of salts like culture medium.

Firstly, 11 types of precisely size-controlled polystyrene spheres (7, 10, 15, 20, 25, 30, 40, 50, 60, 70 or 80 μm in diameter) were placed on a flat silicon substrate, and Ni was deposited on the spheres by thermal deposition with strict control of its thickness set to 2 nm to convey the property of superparamagnetism to the fabricated particles (see also Suppl. Movies S1 and S2). To enhance the magnetic charge of a magcup, we added more Ni layers on it (this time, finally three layers) with spacing material of SiO_2_, with strict control of its thickness at 15 nm (this time, two layers between three layers of Ni), because spacing of less than 15 nm is insufficient to maintain the superparamagnetic properties and the Ni layers change to exhibit ferromagnetic properties. Finally, the Ni/SiO_2_-coated particles were heated at 500°C for 17 h to remove the polystyrene sphere cast; then, magcups were obtained (Figs. 1 (b) and (c)). We confirmed complete removal of polystyrene spheres through immobilizations of biomolecules onto interiors of magcups in our previous studies^18^,^19^, and also confirmed by energy dispersive X-ray spectrometry (EDS) of the FE-SEM analyses as significant decrease of carbon peak in the measurements (Suppl. Fig. S1).

**Figure 2:**
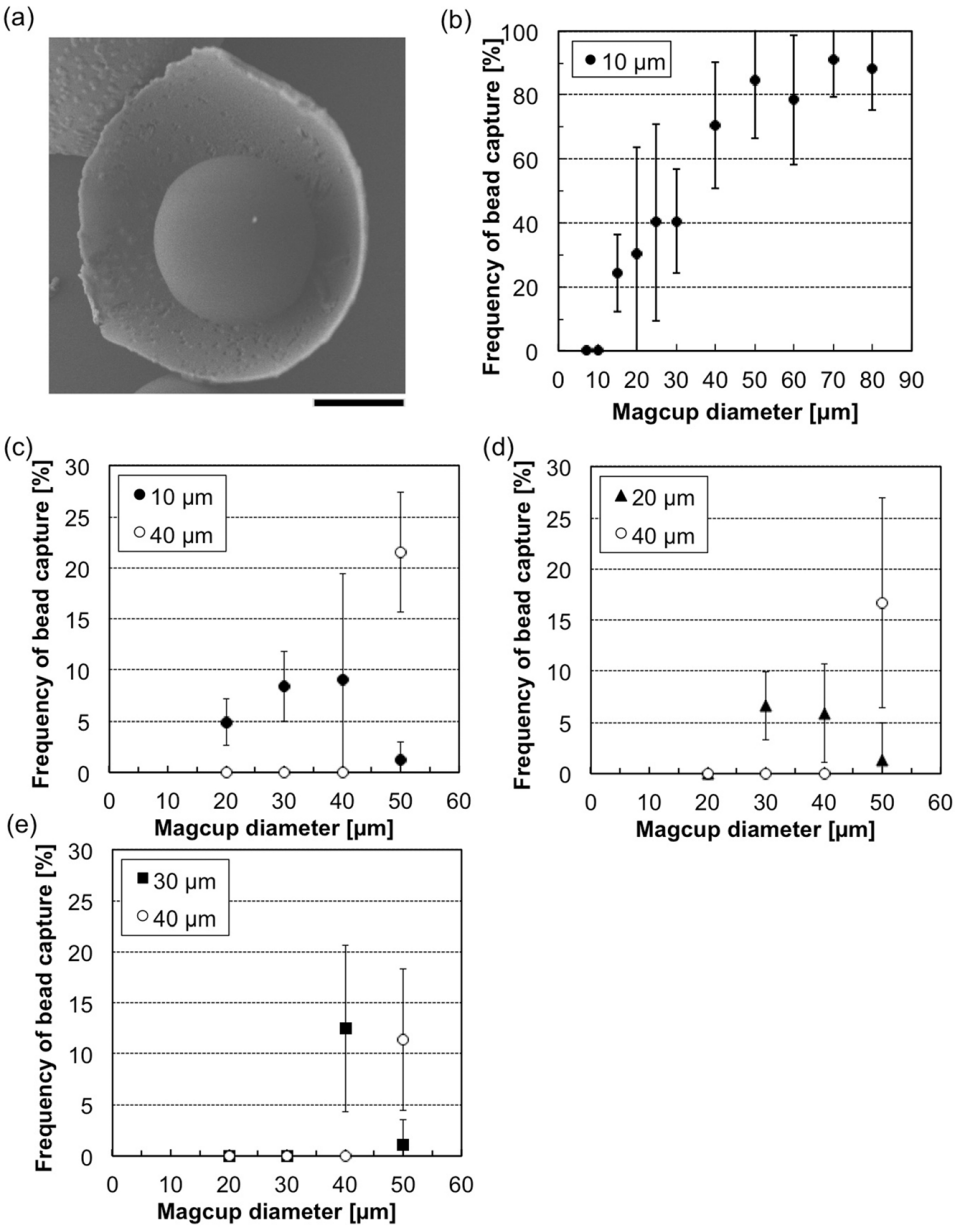
Capture of model target beads using the magcups. (a) An FE-SEM image of a 20-μm magcup in which a 10-μm bead was captured. Bar, 5 μm. (b) Relationship between the frequency of 10-μm-bead-conjugated magcups and the cup diameter. (c–e) Relationship between the frequency of 10- or 40- (c), 20- or 40- (d), and 30- or 40-μm (e) bead-conjugated magcups and the cup diameter. Error bars indicate standard deviations (S.D.).

Subsequently, we examined whether the hemispherical shape of a magcup can be applied for the size filtration of cells. First, size-uniform polystyrene beads of 10 μm in diameter (2 × 10^7^/mL) were pre-coated with bovine serum albumin to enhance binding affinity to the inner surface of the magcups; then, the polystyrene beads were mixed with magcups having an inner diameter of 7, 10, 15, 20, 25, 30, 40, 50, 60, 70 or 80 μm (3 × 10^6^/mL). After 1 h of incubation, the polystyrene beads were successfully captured in the cavity of the magcups as shown in Fig. 2 (a). The number of bead-conjugated magcups was counted for at least 200 cups for each diameter to evaluate the size-selective acquisition ability of the inner cavity of the magcups (Fig. 2 (b)). As shown in the graph, the beads were specifically captured by magcups with diameters equal to and larger than 15 μm, and the frequency of bead-conjugated magcups gradually increased with an increase of the size of the magcup, up to 90% at maximum; however, the magcups smaller than 15 μm, which is the same size or smaller than the polystyrene beads, did not capture any polystyrene beads (0% for both 10-μm (n = 320) and 7-μm magcups (n = 331)). Similar results were acquired in the case of 7-μm polystyrene beads (0% in 7-μm magcups, and 21% in 10-μm magcups) and 15-μm polystyrene beads (0% in 15-μm and smaller magcups). In addition, each suspension of two different size beads having 10 and 40 μm (Fig. 2 (c)), 20 and 40 μm (Fig. 2 (d)), or 30 and 40 μm (Fig. 2 (e)) was mixed with magcups having an inner diameter of 20, 30, 40 or 50 μm to evaluate filtration abilities of magcups. In results, smaller beads than magcup diameters were only captured from the bead suspensions (e.g., 10 μm beads were only collected using 20 μm magcups from 10 and 40 μm bead mixture in Fig. 2 (c)). These results indicate that the inner cavity of the magcup behaves as a size filter to exclude targets larger than the magcup diameter. An exact threshold of size filtration was achieved by uniform size distribution of magcups, with a low coefficient of variation (CV) of less than 3%. For twice and more larger magcups than target beads, a portion of magcups captured more than two beads in a cup with an increase of the frequency of bead-conjugated magcups. For larger magcups than both bead diameters (i.e., 50 μm magcups), large beads (i.e., 40 μm beads) were more frequently captured than small one, might be caused on the depletion effect in which entropic driving forces to conjugate beads with magcups were acted to make a total overlapping volume between beads and magcups as maximum under co-existence of nanoscopic polymers, which yielded frequent acquisition of beads having close diameters with that of magcups^20^,^21^.

**Figure 3:**
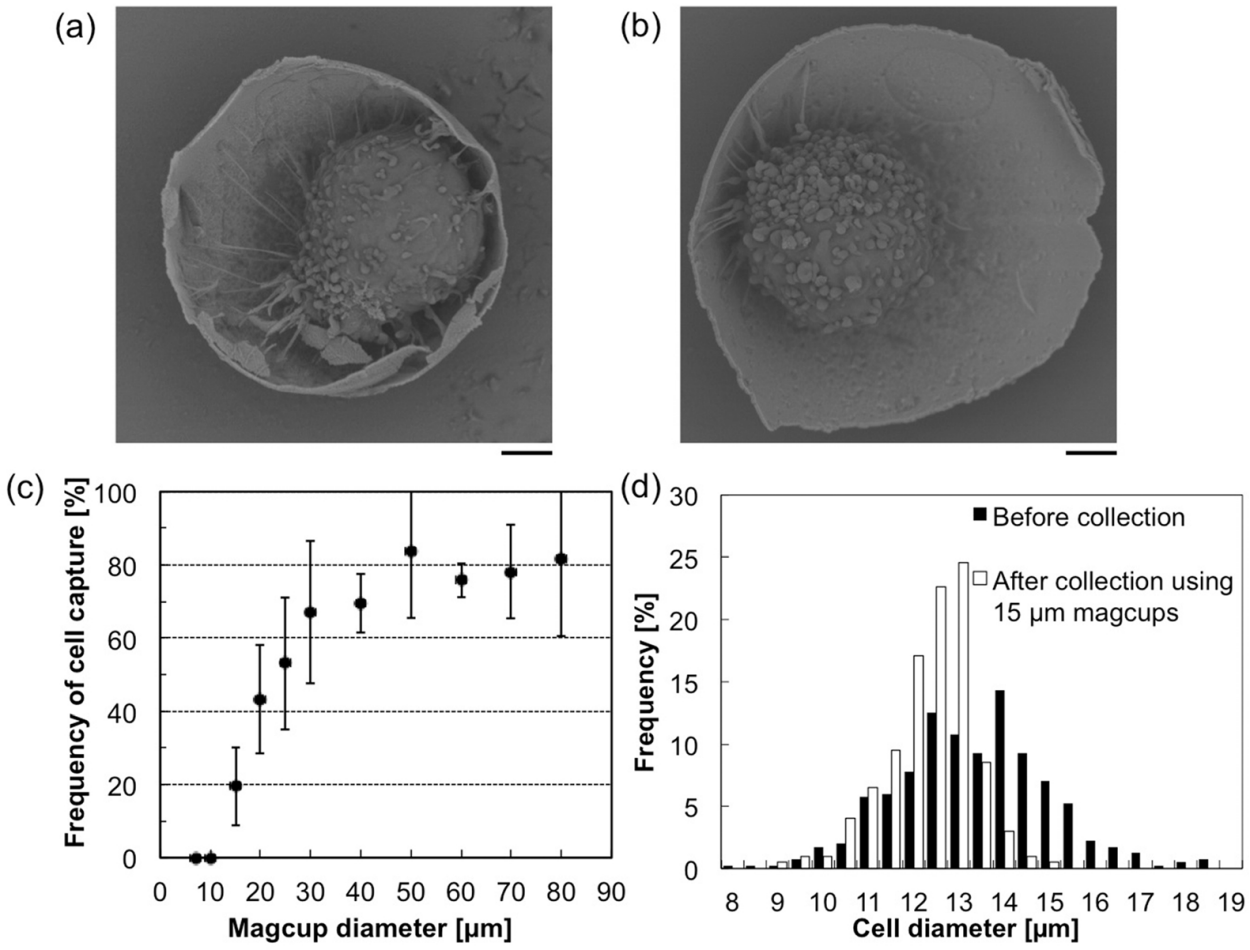
Capture of CCRF-CEM cells using the magcups. (a, b) Typical FE-SEM images of 15- (a) and 20-μm magcups (b) in which the target cells were captured. Bar, 2 μm. (c) Relationship between the frequency of the target-cell-conjugated magcups and the cup diameter. (d) Distributions of cell diameters before and after cell collection assays using 15 μm magcups. Error bars indicate S.D.

We also evaluated size-selective collection of the fabricated magcups for the specific target cells. A size of magcup (any one of the 11 types of magcup having a diameter from 7 to 80 μm) was mixed with human acute lymphoblastic leukemia cells (CCRF-CEM, 12 ± 2 μm in diameter) and incubated for 30 min. Then, the magcups were collected by the application of a magnetic field using a magnet. As clearly shown in the scanning electron micrographs, target cells were successfully captured in the magcup cavities with sizes of 15 μm (Fig. 3 (a)) and 20 μm (Fig. 3 (b)). Almost all cells were specifically attached to the interior of magcups, because a contact area between the cell and a cup exterior is small and therefore, adhesion force is too weak for enough attachment of the cell to the cup exterior. The frequency of cell-conjugated magcups was determined by counting in the same manner as for the previous polystyrene bead collection to evaluate size-selective target filtration (Fig. 3 (c)). From the results, target cells were size-specifically captured by magcups with diameters equal to and larger than 15 μm, but were not captured by 7- and 10-μm magcups. Diameters of captured cells using 15 μm magcups were minutely measured (n = 200) and the distribution was compared with that before the collection assays (n = 400). Figure 3 (d) shows the result of comparison, and as shown in the figure, cells smaller than 15 μm were only purified by the magcup collection assays. These results are consistent with that in the previous polystyrene bead collection; namely, the magcups only collect target cells that are smaller than their inner cavity.

**Figure 4:**
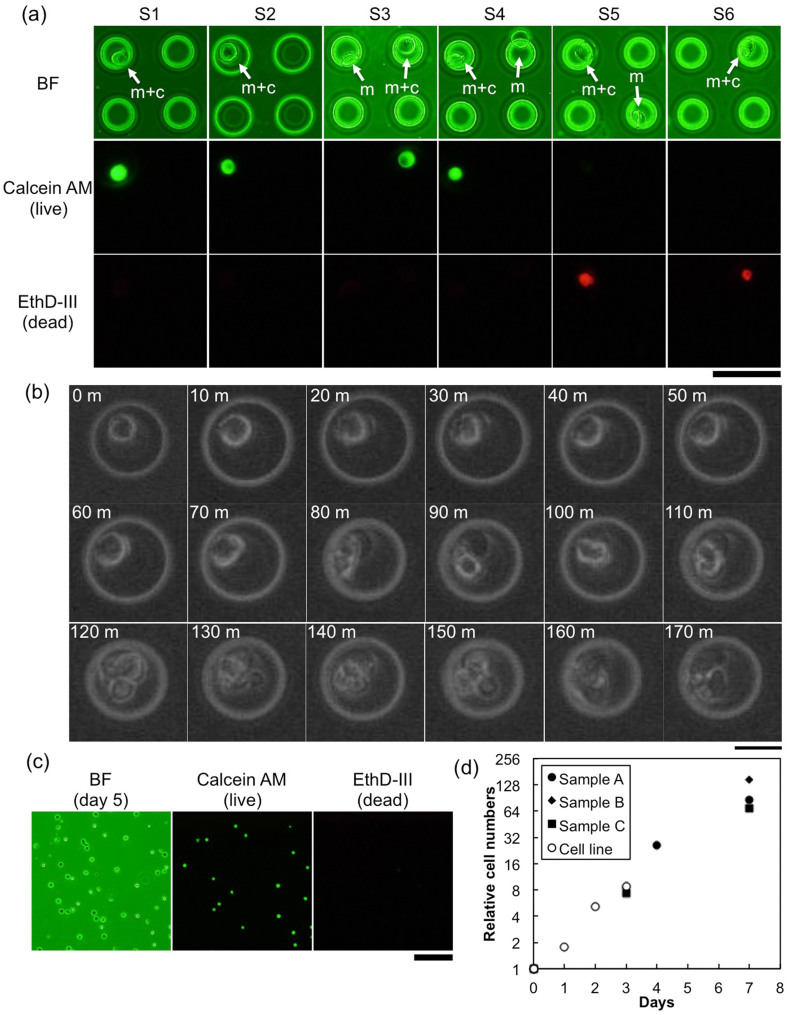
Evaluation of collected cell survival. (a) Optical and fluorescent microscope images of two-color fluorescent staining results for six typical samples (S1–S6). Cells captured by the magcups (m + c) and empty magcups (m) are indicated by arrows in bright field (BF) images. Bar, 50 μm. (b) Time-lapse observation of a collected cell. The cell still in the magcup was placed into a small PDMS chamber (shown as an outer white circle in the pictures) to prevent diffusion, and pictures were taken every 10 min. The time elapsed since the start of monitoring is shown in each picture. Bar, 20 μm. (c) Optical and fluorescent microscope images of two-color fluorescent staining results for collected cells using magcups after 5 days of incubation in culture medium. Bar, 100 μm. (d) Relationships between relative cell numbers and the periods elapsed since the start of cultivation for three independent cell collection assays by the magcup (black circle, diamond, and square). Results for a cultivated CCRF-CEM cell line are also plotted (white circle).

After the collection of target cells using the magcups, the survival of collected cells was evaluated. Collected cells trapped in the magcups were placed into small incubation chambers made of poly(dimethylsiloxane) (PDMS) on a cover slip to prevent their diffusion, and the survival was evaluated using two-color fluorescent staining of live (Calcein AM, green) and dead cells (EthD-III, red). Figure 4 (a) shows optical and fluorescent microscope images of the staining results for six typical samples (S1–S6). As shown in the figure, cells captured by the magcups (indicated as “m + c”) were stained either green (live) or red (dead), but empty magcups (indicated as “m”) were not stained for both colors. The survival rate was 87%, which is almost the same as that before the collection assay, 93%, indicating that the damage associated with the collection assay was almost negligible. Next, collected cells were incubated in cell culture medium with time-lapse observation. Figure 4 (b) shows typical sequential optical bright field micrographs of the time-lapse observation taken every 10 min (the movie is also available online as Suppl. Movie S3). The cell, which might have been ready to enter the mitosis phase, first escaped from its magcup 80 min after the start of the observation, and 120 min after the start, it divided into two cells. After incubation of collected cells with magcups, cell survival was evaluated again. Figure 4 (c) shows optical and fluorescent microscope images of the staining results by the same procedure as in Fig. 4 (a) after 5 days of incubation of collected cells. As shown in the pictures, collected cells survived even though they were incubated with the magcups. The survival rate was 91%, which also indicated almost negligible damage associated with collection assays using magcups. The growth curves of collected cells were also determined at a bulk scale to evaluate their level of damage. In this evaluation, the numbers of cells were normalized by the number upon initial collection to avoid bias due to differences in collected cell number in each assay. Figure 4 (d) shows the results of cell increase for three independent collection assays with a curve of increase for the cultivated CCRF-CEM cell line. As shown in the figure, gradients of cell increases were almost the same as that of the cultured cell line, approximately doubling each day. These results clearly indicate that collected cells survived without serious damage, detached from the inner cavity of the magcup without any treatment, and could be re-cultivated in culture medium.

## Discussion

In this study, precisely size-controlled magcups were fabricated and used to collect desired target cells depending on their sizes as a physical shape biomarker. We compared the developed magcup method with popular conventional methods, e.g., MACS and membrane filtration. There are five important viewpoints for cell purification, i.e., cell size filtration, molecular biomarker-based cell collection, combined purification of size and molecular biomarker, batch-treatment (or high-throughput treatment), and non-invasive (damage-free) cell collection, and the magcup method can cover all these five characteristics. As MACS is one of the most popular and powerful methods for expressed target molecule-based cell purification, that all cells on which target biomarker molecules are expressed are collected with recognition and target-antibody interaction by antibodies immobilized on the magnetic particles regardless of their sizes. Therefore, this method lacks the ability of size-filtration function. In contrast, the membrane filtration can provide size filtration by controlling pore size on the membrane. However, strong shear stress which is applied to cells during the filtration sometimes gives serious damage to cells, or it might give some clotting of cells on the membrane surface^22^,^23^,^24^. Upon comparison of these methods, we can regard our method as an advanced MACS method which can cover size filtration function with maintaining all advantages of conventional MACS method, and our method can overcome all existing problems in conventional membrane filtration method. In addition, our method can combine molecular filtration with size filtration at the cell purification. Moreover, the addition of size filtration function to the MACS method can give a potential to achieve more reliable purification of target cells by avoiding collection of clustered cells, in which undesired cells are contaminated.

In this study, magcups were composed of stacked Ni and SiO_2_ layers to add superparamagnetic properties for the cups. There are some indications about the toxicity of Ni for living systems based on *in vitro*^25^ and *in vivo*^26^ examinations. For studies *in vitro*, incorporation of Ni compounds into the cell could be harmful in terms of the maintenance of normal functions of the living system; therefore, dissolution of Ni compounds should be taken into account. Ni is in general water-insoluble and, in fact, the magcups used in this study retained their shape completely even though they were exposed to solvents in order to perform cell collection assays. Moreover, collected cells survived even when they were cultivated with magcups for a long period, as shown in Fig. 4, indicating low toxicity of the magcups for target cells in the *in vitro* assays.

In conclusion, various sizes of fabricated superparamagnetic magcup can be used as a tool for cell filtration and collection, which is as convenient as conventional MACS, adding the function of cell-size filtration, which is a major advantage of the magcups as a useful target cell collection tool.

## Methods

### Fabrication of cup-shaped superparamagnetic hemispheres

The superparamagnetic hemispheres (referred to as “magcups” in the text, and also hereafter) were fabricated using polystyrene spheres as templates by the following procedures^13^,^14^,^15^,^16^,^17^,^18^. First, commercially available suspensions of polystyrene spheres of 7 to 80 μm in diameter (DYNOSPHERES; nominal diameters, 7.088, 10.14, 15.62, 20.31 and 25.01 μm; coefficients of variation (CV), 0.80, 1.20, 1.17, 1.17 and 2.98%; JSR, Japan; and Duke Standards, nominal diameters, 29.75, 39.94, 50.2, 59.2, 69.1 and 79.0 μm, and CV, 1.4, 1.3, 1.0, 1.4, 1.2 and 1.3%, respectively; Thermo Scientific, CA, USA) were dropped onto clean flat silicon (Si) substrates as one diameter of the sphere on a substrate, and dried. Next, the polystyrene spheres were placed into a vacuum evaporator (VPC-1100, ULVAC, Japan) to coat elements on their surfaces, and nickel (Ni) and silicon dioxide (SiO_2_) were sequentially coated on the spheres alternately and as thin enough to convey superparamagnetism, typically 2 nm for Ni and 15 nm for SiO_2_. Finally, the element-coated spheres were placed into an electric furnace (MMF-2, Asone, Japan) and incubated at 500°C for 17 h to remove the polystyrene sphere templates. To use the obtained magcups in dispersed states, a suitable solvent, typically ultra-pure water containing 0.1% Tween 20, was dropped on a Si substrate attached to the magcup, and the cup was dislodged from the substrate by the gentle application of ultrasound in a sonicator; then, the cup was dispersed in the dropped solvent.

### Collection of polystyrene beads using the magcups

For the bead collection using the magcups, bovine serum albumin (BSA)-coated fluorescent polystyrene beads (Fluoresbrite™ carboxylate YG microspheres, nominal diameter of 10.08 ± 0.97 μm; Polyscience, USA) were used as a model target. The BSA molecules were coated to allow non-specific binding of the beads to the magcup surfaces. For the immobilization of BSA onto the bead surface, 2 × 10^6^ carboxylated beads were activated with 10 mg/mL 1-ethyl-3 (3-dimethylaminopropyl) carbodiimide hydrochloride (EDC, Thermo Scientific, USA) and 10 mg/mL N-hydroxysulfosuccinimide (NHS, Wako, Japan) in pH 5.0 2-morpholinoethanesulfonic acid (MES) for 15 min at room temperature, washed with MES 3 times, reacted with 1% BSA (Life Technologies, USA) in pH 7.4 phosphate-buffered saline (PBS) for 1 h at room temperature, and washed with PBS 3 times. Then, 3 × 10^5^ magcups of various diameters (i.e., 7, 10, 15, 20, 25, 30, 40, 50, 60, 70 or 80 μm) were reacted with the BSA-coated beads in ultra-pure water containing 0.1% Tween 20 for 1 h at room temperature. After the reaction, the sample was washed with the same kind of solvent 3 times and observed using a fluorescent microscope to count the number of bead-conjugated magcups.

For the collection of two different size beads, polystyrene beads (diameters, 10.14, 20.31, 29.75 and 39.94 μm, CV, 1.20, 1.17, 1.4 and 1.3%, respectively, JSR and Duke) were mixed with 1% BSA in pH 7.4 PBS for 1 h at room temperature, washed with PBS 3 times, and adjusted as two bead volumes as the same in the solvent at 0.1% (v/v) concentrations. The suspensions were reacted with 20, 30, 40 or 50 μm magcups as the same procedure with the above single size bead collection assays.

### Collection of target cells using the magcups

For the collection of target cells using the magcups, a human acute lymphoblastic leukemia cell line (CCRF-CEM) was chosen as a model target, and was maintained in culture medium at 37°C and under 5% CO_2_ in RPMI 1640 (Life Technologies) supplemented with 10% heat-inactivated fetal bovine serum (Asahi Glass, Japan) and 100 U/mL penicillin-100 μg/mL streptomycin (Life Technologies). In the reaction, 1 × 10^6^ cells were reacted with 3 × 10^5^ magcups in binding buffer^27^, consisting of Dulbecco's PBS containing 25 mM glucose, 5 mM MgCl_2_, and 1% BSA, for 30 min at room temperature with gentle mixing every 10 min. After the reaction, the sample was washed with binding buffer 3 times, dropped on a glass, and observed using an optical microscope to count the number of cell-conjugated magcups. For the measurement of collected cell diameters, the optical microscope images were taken and the diameters were calculated using image analysis software (Image J 1.48, http://imagej.nih.gov/ij/).

### Re-cultivation of target cells collected with the magcups

To evaluate the survival of the cells collected using the magcups, time-lapse observation of the cells was performed. Firstly, a small poly(dimethylsiloxane) (PDMS) chamber array was fabricated in a cell culture dish as follows. The PDMS (SYLGARD 184 silicon elastomer, Dow Corning, USA) sol was dropped onto a Si mold on which microcolumns (30 μm in diameter and 30 μm in height) had been pre-fabricated. The dropped PDMS sol was heated at 90°C for 1 h to harden it. After heating, the PDMS was then peeled off from the mold and attached on a glass-based cell culture dish (Asahi Glass).

The cells collected using the magcups were placed into the PDMS chamber while still captured in the magcup and a few milliliters of cell culture medium was carefully added to the dish. The dish was set on an inverted microscope (IX-71, Olympus, Japan) combined with both a stage top incubator (Tokai Hit, Japan) and a time-lapse CCD acquisition system. Images of the re-cultivated cells were then taken every 10 min.

For two-color fluorescent staining of live and dead cells, a commercially available live/dead cell staining kit (PromoCell, Germany) was used. The kit contained two fluorescent probes, Calcein AM (live cell staining in green) and EthD-III (dead cell staining in red), and those probes were adjusted to 10 μM using PBS. The probe solution was applied to the collected cells still captured in the magcups, and incubated for 30 min at room temperature. After the incubation, the cells were washed with PBS and microscopic observations were performed.

### FE-SEM observation

The results of the magcup reactions were confirmed by direct observation of the cup using field emission scanning electron microscopy (FE-SEM; JSM-6701F, JEOL, Japan) with a backscattered electron (BSE) detector (SM-74071, JEOL). In the case of cell-conjugated magcup observation by FE-SEM, the reacted sample was immersed in 70, 80, 90, 95 and 99% ethanol sequentially for dehydration. The cells were additionally immersed in 100% ethanol, which was dehydrated with copper sulfate, twice, and then tert-butyl alcohol, and incubated at −20°C for 1 h. The specimen was placed in a freeze-dry system (Labconco, USA) and freeze-drying was performed. Before the FE-SEM observation, samples were coated with platinum using an automatic fine coater (JFC-1600, JEOL) with a sputtering current of 30 mA for 40 s. The observation conditions were as follows: 5 kV acceleration voltage, ×1,000 to ×5,000 magnification, 200 pA probe current, 8 mm working distance, 20 s capture time, 1280 × 1024 pixels in a picture, and secondary electron (SE) and BSE detection mode. An incident electron beam was vertically applied to the sample surface, and the BSE was monitored with its scattering angle ranging between 16° and 60° from the axis of the incident electron beam.

## Author Contributions

H.K. designed experimental conditions throughout the study, and performed measurements and data analysis. H. Terazono contributed to determine experimental conditions for cell capture, time-lapse observation and re-cultivation of collected cells, and data processing throughout the study. H. Takei conceived the fabrication method of Mag cups and conducted the particle fabrication. K.Y. conceived and conducted the design of this study. The manuscript was written by H.K. and K.Y.

## Supplementary Material

Supplementary Information Supplementary Information for Readers

Supplementary Information Supplementary Movie S1

Supplementary Information Supplementary Movie S2

Supplementary Information Supplementary Movie S3
