# Supplementary material for: Cup-Shaped Superparamagnetic Hemispheres for Size-Selective Cell Filtration
Source: Sci Rep. 2014 Sep 15;4:6362. doi: 10.1038/srep06362 (PMC4163672; doi:10.1038/srep06362)
Supplement: Supplementary Information — for Readers [file srep06362-s1.pdf]

Supplementary Information

Cup-Shaped Superparamagnetic Hemispheres for Size-Selective Cell Filtration

Hyonchol Kim, Hideyuki Terazono, Hiroyuki Takei, Kenji Yasuda*

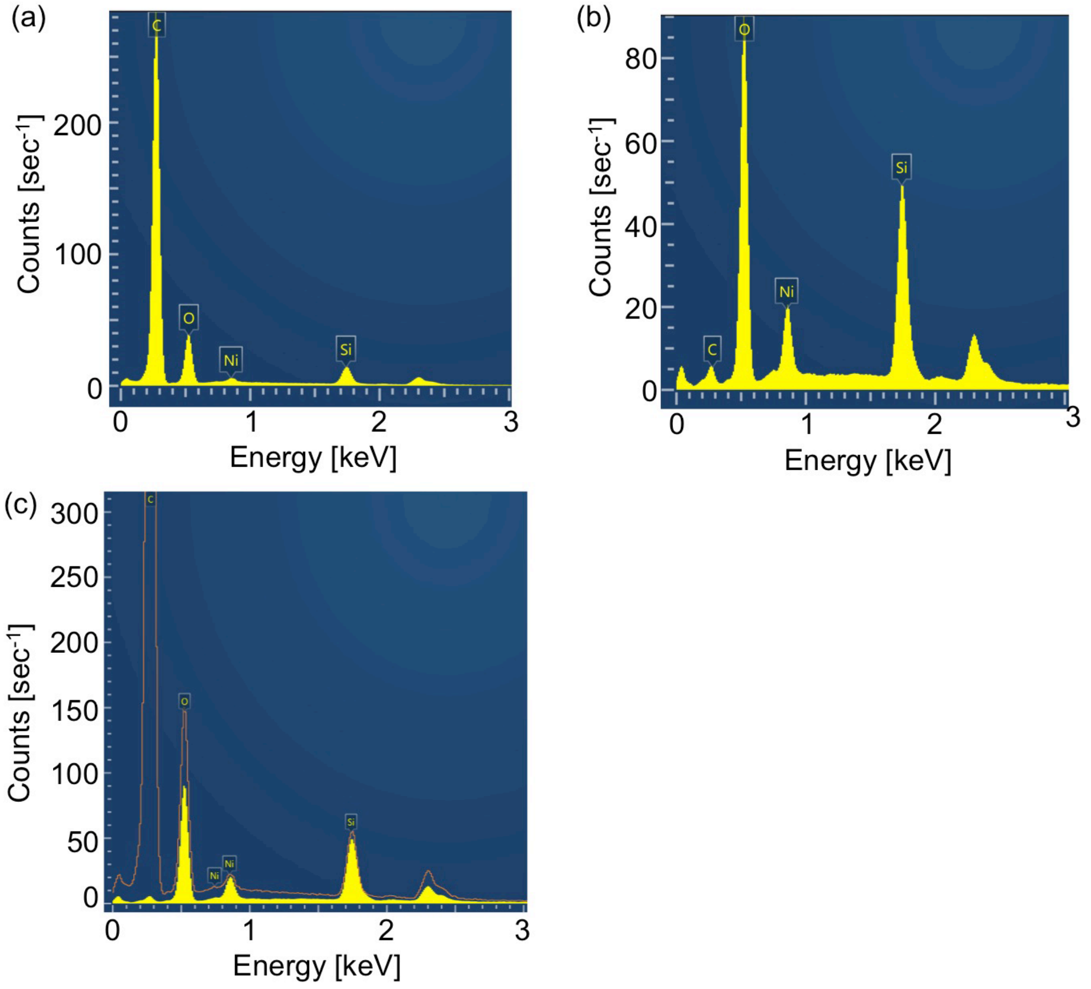

Supplementary Figure S1

Energy dispersive X-ray spectrometry (EDS) analyses for magcups before (a) and after (b) burning process at their fabrication procedures to remove polystyrene spheres from cup interiors. (c) Overlap of two spectra. Solid line, before (a), and filling, after (b) the burning process.

Supplementary Movies S1 and S2

Collection of the fabricated magnetic particles using a neodymium magnet. The Ni-coated particles with a thickness of either 2 nm (Movie S1) or 5 nm (Movie S2) were dispersed in ultra-pure water containing 0.1% Tween 20 before polystyrene burning, placed on a glass slide, and encapsulated with another glass using a spacer of 0.3 mm thickness; then, a static magnetic field was applied by placing the magnet on the particle-encapsulated glass. The magnet is shown as a circular silhouette in the movies.

Supplementary Movie S3

Time-lapse observation of collected cells using magcups. Cells still in magcups were put into a small PDMS chamber array (shown as white circles, 30 μm in diameters) to prevent diffusion, pictures were taken every 10 min, and the movie was constructed as each picture corresponded to 1 s.
